# Knowledge Sharing in AI-Enabled Workplaces: A Social Cognitive Perspective on Usefulness Perceptions and Competition

**DOI:** 10.3390/bs15121635

**Published:** 2025-11-27

**Authors:** Chuling Zhong, Yao Wang

**Affiliations:** School of Business, China University of Political Science and Law, Beijing 100088, China

**Keywords:** artificial intelligence, knowledge sharing, social cognitive theory, digital transformation, organizational climate

## Abstract

The integration of artificial intelligence (AI) into organizational processes brings both opportunities and challenges for knowledge sharing. Knowledge sharing remains a cornerstone of organizational learning and innovation, yet the emergence of AI introduces new complexities to this behavior. In particular, AI-related knowledge is both a valuable tool and a controversial technology, whose dual nature generates uncertainty in employees’ evaluations and decisions about sharing it. Drawing on social cognitive theory (SCT), this study develops a moderated mediation model to explain how personal cognitions and environmental cues jointly shape AI-related knowledge sharing. Within the SCT framework, perceived AI usefulness represents a personal cognitive evaluation, whereas anticipated positive response reflects a social outcome expectation. When employees perceive AI as highly useful, they are more likely to expect positive social feedback, which in turn motivates AI-related knowledge sharing behavior. Moreover, organizational competitive climate weakens this effect by acting as a social discount factor that reduces the perceived significance of social rewards. A three-wave survey of 519 Chinese employees supports these hypotheses, extending SCT by revealing how the dual nature of AI shapes employee outcome expectations and by identifying a socially grounded cognitive pathway that links perceived AI usefulness to knowledge sharing behaviors in organizational contexts.

## 1. Introduction

AI technology, with its capabilities in automating tasks, improving decision-making, and facilitating other progress, has shown significant potential in enhancing the performance of certain jobs (Morandini et al., 2023; X. Yu et al., 2023; Olan et al., 2022; Hassani et al., 2020). However, mastering AI technology requires specific resources and expertise, such as effective system scaffolding and feedback (Wisniewski et al., 2020), computational resources and background knowledge (Cetindamar et al., 2022), and hands-on experience with AI (Li & Kim, 2024). These requirements serve as barriers to developing AI skills, leading to disparities in AI proficiency among employees within an organization (Cetindamar et al., 2022). In this context, encouraging employees with AI expertise to share their knowledge is critical for improving their colleagues’ AI skills and enhancing organizational effectiveness in the AI era (Chowdhury et al., 2022; Binsaeed et al., 2023).

Prior research has identified various antecedents of knowledge sharing, including individual perceptions and organizational contextual factors (S. Wang & Noe, 2010). However, because of the distinctive nature of AI technology, the determinants of employees’ AI-related knowledge sharing may be more complex. Unlike traditional tools designed for specific tasks, AI is a transformative and fast-evolving technology that reshapes workflows and generates new insights across industries (Hassani et al., 2020), heightening employees’ perceptions of its usefulness. At the same time, AI’s rapid evolution raises concerns about accuracy, applicability, and usage specification, making it a source of professional and social controversy (Cortiñas-Lorenzo et al., 2024; Osasona et al., 2024; Zhou et al., 2024). On one hand, AI-related knowledge represents a valuable resource that allows employees to demonstrate expertise and contribute to collective performance, motivating them to share it. On the other hand, the same knowledge can be seen as a scarce source of competitive advantage or as a potentially risky and controversial tool, prompting employees to withhold it to protect personal interests or avoid accountability for negative outcomes. These opposing forces introduce uncertainty about the social and professional outcomes of sharing AI-related knowledge. Yet, existing research has offered limited explanation of how employees navigate this uncertainty when deciding whether to share AI knowledge.

To explain how employees make knowledge sharing decisions under such uncertainty, this study draws on social cognitive theory (SCT), which posits that individual behavior results from the reciprocal interaction among personal, behavioral, and environmental factors (Bandura, 1986). Within this framework, personal perceptions and expectations are key determinants of motivation (Schunk & DiBenedetto, 2020). For instance, employees’ perceptions of the usefulness of skill learning (such as improving job performance) have been shown to shape their expectations of desirable outcomes (such as career advancement or higher pay), which in turn encourages participation in developmental activities (Colquitt et al., 2000). Extending this logic to AI-enabled workplaces, two cognitive mechanisms become particularly salient: perceived AI usefulness, reflecting employees’ evaluation of how effectively AI enhances their work performance, and anticipated positive response, denoting their expectation of being recognized or appreciated by colleagues for sharing AI-related knowledge. When employees perceive AI as highly useful, they are more likely to expect that sharing their AI expertise will generate favorable social responses—such as acknowledgment, gratitude, or respect from peers—thus strengthening their motivation to engage in AI-related knowledge sharing.

Social cognitive theory further emphasizes that cognitive processes do not operate in isolation but are shaped by environmental influences that guide how individuals interpret and respond to social information (Bandura, 1986). Building on this premise, the organizational competitive climate represents a critical contextual cue that affects how employees evaluate the potential costs and benefits of sharing knowledge. In highly competitive environments, strong social comparison and status pressure increase the perceived personal costs of disclosure, while diminished trust and reciprocity reduce the credibility and possibility of positive feedback. Under such conditions, employees may discount or even doubt the significance of social rewards, weakening the motivational link between perceived AI usefulness and anticipated positive response. Conversely, in less competitive and more collaborative climates, reduced status pressure and stronger interpersonal trust create a psychologically safe environment for sharing. Employees in such contexts are more confident that their efforts to share AI knowledge will be understood and valued, reinforcing their expectations of favorable social outcomes.

**Figure 1:**
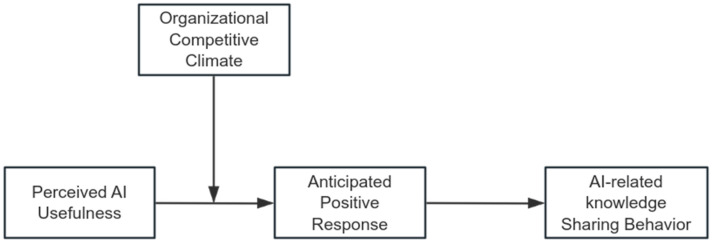
Research model.

To test these hypotheses, our study constructed a moderated mediation model and collected multi-wave survey data from 519 employees in China. The conceptual model and hypothesized relationships are illustrated in Figure 1. Through empirical analysis, we aim to contribute to both theoretical and practical discussions on AI knowledge sharing behavior by examining how perceived AI usefulness, anticipated positive response and organizational competitive climate interact to shape it. We hope our findings can provide new insights into promoting knowledge sharing in AI-integrated workplaces and offer strategic guidance for organizations navigating AI-driven transformations.

The remainder of this paper is structured as follows. Section 2 reviews the theoretical background and develops our hypotheses. Section 3 outlines the research design, including the sample, measures, and analytical strategy. Section 4 presents the empirical results. Section 5 discusses the theoretical and practical contributions, addresses the study’s limitations, and outlines directions for future research.

## 2. Theory and Hypothesis Development

### 2.1. Social Cognitive Theory

Social cognitive theory is a widely accepted model for explaining individual behavior (Compeau & Higgins, 1995). In the SCT model, personal factors and environmental factors jointly influence individuals’ behavior. Specifically, individuals cognitively evaluate both the environment and themselves, form outcome expectations of their actions based on this assessment, and then adopt behaviors accordingly (Bandura, 1999). In the field of management, SCT provides a key framework for explaining and predicting individual work behavior, with recent studies applying it to career self-management (S. D. Brown & Lent, 2019), knowledge sharing within project teams (Ren & Sun, 2024), and proactive service behavior (Zhan et al., 2025). By integrating cognitive and situational factors, SCT offers a comprehensive approach to understanding workplace behavior.

From the perspective of SCT, knowledge sharing is a cognitively driven behavior shaped by personal cognition and environmental cues (Hsu et al., 2007). In this study, perceived AI usefulness and anticipated positive response are both conceptualized as personal factors: the former reflects employees’ cognitive appraisal of AI’s value in improving work performance, while the latter captures their anticipated social outcomes from engaging in knowledge sharing. Organizational competitive climate represents the environmental factor, indicating the extent to which interpersonal interactions and recognition are influenced by internal competition. Finally, AI-related knowledge sharing behavior constitutes the behavioral factor, reflecting employees’ actual engagement in knowledge exchange. Together, these components illustrate how personal cognition, environmental context, and behavior interact within the SCT framework to shape knowledge sharing decisions in AI-integrated workplaces.

### 2.2. Perceived AI Usefulness and Knowledge Sharing Behavior

Knowledge sharing behavior refers to the provision of task information and know-how to help others and to collaborate with others to solve problems, develop new ideas, or implement policies or procedures (S. Wang & Noe, 2010). In the work context, knowledge is defined as the information processed by individuals including ideas, facts, expertise and judgments relevant for individual, team, and organizational performance (S. Wang & Noe, 2010; Akram et al., 2020). In today’s workplace, where knowledge workers play an increasingly critical role, the knowledge they proactively share helps organizations accumulate intellectual capital, supports employee growth, and enhances overall performance (O’Neill & Adya, 2007). Such knowledge sharing fosters problem-solving, drives innovation, and supports the dissemination of best practices, all of which are important for organizations to adapt to rapidly changing environments and maintain a sustainable competitive advantage (Nonaka et al., 1996; Z. Wang & Wang, 2012; Castaneda & Cuellar, 2020).

Under what circumstances are employees more willing to share their knowledge proactively? According to the SCT, when individuals believe they are capable of performing a task and achieving positive outcomes, they are more likely to engage in that behavior; conversely, if they doubt their ability, they may be less inclined to take action (Bandura, 1982, 1986; Igbaria & Iivari, 1995; Schunk & DiBenedetto, 2021). For example, Kankanhalli et al. (2005) have shown that employees who are confident in their knowledge and abilities are more willing to share insights with others and contribute to the organization. Thus, from the perspective of SCT, if employees perceive their shared knowledge as useful, such as solving job-related problems (Constant et al., 1996), helping their colleagues (Kankanhalli et al., 2005), or make a difference in their organization (Kollock, 1999; Wasko & Faraj, 2000), they are more likely to engage in knowledge sharing behavior.

AI broadly refers to intelligent support systems built on algorithms, natural language processing, machine learning methods, and human intelligence, capable of learning, interacting, problem-solving and so on (Akkiraju et al., 2006). In recent years, AI has been increasingly applied in modern organizations, including but not limited to supporting decision-making, automating repetitive tasks, and enhancing information analysis (Raisch & Krakowski, 2021). Therefore, AI-related technologies, experiences, and information have become highly valuable knowledge resources in today’s work context, attracting increasing attention for their practical usefulness (Olan et al., 2022).

Drawing on Davis’s (1989) definition of perceived usefulness in the context of technology, we define perceived AI usefulness as employees’ perception of AI technologies’ ability to enhance various aspects of their work. We propose that when employees perceive AI as useful, they are more likely to expect AI knowledge sharing to lead to positive outcomes. For example, they may anticipate that the AI knowledge they share will help colleagues solve complex work challenges (Memmert & Bittner, 2022; Johnson et al., 2022). Alternatively, they may expect that their contributions of AI knowledge will lead to innovation and breakthroughs for the organization (Mariani et al., 2023; Haefner et al., 2021). These beliefs increase their confidence in their knowledge sharing behavior—that is, they believe the knowledge they share can make a difference at work, thereby strengthening their motivation to share AI-related knowledge with others.

Following this theoretical framework, we propose the following hypothesis:

**H1.**  *Perceived AI usefulness positively influences employees’ AI-related knowledge sharing behavior.*

### 2.3. Anticipated Positive Response as a Mediator

Beyond the belief that useful AI-related knowledge can enhance work performance, employees’ perceptions of AI usefulness also foster proactive knowledge sharing behavior by strengthening their anticipation of positive response. SCT suggests that an individual’s behavioral motivation can be regulated by outcome expectations regarding social effects (Bandura, 1997). Anticipated positive response represents a specific form of social outcome expectation—that is, individuals’ cognitive anticipation of favorable emotional or relational reactions from others following their behavior. Unlike general intrinsic motivation or extrinsic motivation, anticipated positive response reflects the affective and interpersonal dimension of motivation, emphasizing the social approval employees expect from their peers (Exline et al., 2004).

The perception of AI usefulness significantly influences employees’ expectations of receiving positive feedback from knowledge sharing behaviors. As AI increasingly becomes a strategic asset (Olan et al., 2022), employees who perceive AI as useful tend to believe that sharing AI-related knowledge not only helps improve colleagues’ job performance but is also likely to be regarded as a valuable contribution to organizational success. This, in turn, leads them to expect that their efforts will be emotionally appreciated by peers—eliciting expressions of gratitude, recognition, and appreciation (Imran et al., 2025). Moreover, by providing useful knowledge, employees may position themselves as competent and resourceful individuals within the organization (Nguyen et al., 2021), which in turn fosters the expectation that others will respond with greater trust, respect, and acknowledgment. At the same time, such behaviors may also reinforce expectations of reciprocity, as employees anticipate that their knowledge-sharing efforts will be returned in future interactions with colleagues (Cropanzano & Mitchell, 2005). Collectively, these cognitive appraisals jointly shape employees’ anticipation of positive social feedback.

The anticipation of positive feedback, in turn, serves as a powerful motivator for employees to actively engage in AI-related knowledge-sharing behaviors. Within organizational settings, the pursuit of a strong professional reputation, favorable peer evaluations, and positive workplace relationships are key drivers of employee motivation (Carrillo et al., 2004; Perumal & Sreekumaran Nair, 2022). Given the inherent complexity and uncertainty of AI, the anticipation of positive feedback is important to employees because it provides psychological reassurance that their efforts will be recognized and valued. Moreover, this expectation reduces the perceived risks associated with AI-related knowledge-sharing, such as being judged, ignored, or losing face (Hao et al., 2022). When employees foresee appreciation or constructive responses from peers, they are more likely to interpret knowledge sharing as a high-reward, low-risk behavior. This positive anticipation not only boosts confidence but also strengthens their motivation to contribute, making engagement in AI-related knowledge-sharing more appealing and sustainable.

Given these relationships, it is reasonable to propose that anticipated positive response serves as a mediating mechanism between perceived AI usefulness and employees’ AI-related knowledge sharing behavior. When employees perceive AI as useful, they anticipate that sharing knowledge about AI will elicit positive responses from colleagues. Then, this expectation fosters their willingness to engage in knowledge sharing activities. Consequently, we hypothesize the following:

**H2.**  *Anticipated positive response mediates the relationship between perceived AI usefulness and employees’ AI-related knowledge sharing behavior.*

### 2.4. The Moderating Role of Organizational Competitive Climate

The effect of perceived AI usefulness on anticipated positive response does not occur in isolation—it is shaped by the social environment in which employees work. According to Social Cognitive Theory (Bandura, 1986), individuals’ expectations of social outcomes are also influenced by environmental cues that shape how they interpret and respond to social information. Extending the psychological process proposed in Hypothesis 2, we consider how contextual conditions influence employees’ expectations of social feedback. One particularly important factor is the organizational competitive climate, which reflects the extent to which rewards and recognition depend on comparisons with peers (S. P. Brown et al., 1998), thereby shaping how employees evaluate the potential costs and social value of sharing their AI-related knowledge.

In highly competitive environments, employees operate under conditions of rivalry, social comparison, and uneven reward distribution (David et al., 2021). In such contexts, AI-related expertise is often viewed as a strategic power asset that enhances one’s distinctiveness and professional influence (Jia et al., 2025). Sharing this type of knowledge may thus feel like surrendering a personal advantage or inviting evaluation and imitation by peers, heightening both psychological and strategic risks. At the same time, intense competition undermines the trust and openness that normally facilitate reciprocity and cooperation (J. Yu et al., 2024; Ferrin et al., 2007). Expressions of gratitude or recognition become less frequent and may appear insincere, reducing the perceived possibility and credibility of social rewards. The complexity of AI further amplifies these concerns (Osasona et al., 2024), as employees may fear being misunderstood or criticized for their insights (Rezaei et al., 2024). Consequently, even when employees recognize AI’s usefulness, they may discount the potential benefits of positive social feedback, perceiving it as uncertain or insufficient to offset the tangible risks of knowledge disclosure. Together, these heightened personal costs and diminished social rewards lower the motivational salience of anticipated positive response, thereby weakening the positive relationship between perceived AI usefulness and expected social outcomes.

Conversely, in less competitive and more collaborative climates, interpersonal conflicts and status-driven comparisons are less prevalent, creating an environment of mutual trust and psychological safety (Wu et al., 2021). Employees in such settings tend to view AI-related knowledge as a shared resource that benefits both individuals and the collective (Edmondson, 1999). The reduced pressure of comparison lowers the perceived personal risks of disclosure, allowing employees to focus on the developmental and cooperative value of knowledge sharing. Acts of sharing are interpreted as collaborative and socially valued rather than self-threatening. Peers respond with genuine appreciation, recognition, and constructive feedback, making social rewards more predictable and emotionally meaningful (McEvily et al., 2003). This stability in interpersonal exchange reinforces employees’ confidence that their contributions will be understood and valued rather than misinterpreted or exploited. Consequently, employees perceive social feedback as a credible and rewarding outcome of sharing useful AI knowledge, strengthening the motivational link between perceived AI usefulness and anticipated positive response.

Based on the above analysis, we hypothesize:

**H3.**  *Organizational competitive climate negatively moderates the relationship between perceived AI usefulness and anticipated positive response, such that the relationship is weaker in high-competitive climates and stronger in low-competitive climates.*

Building on these insights, we propose a moderated mediation model in which organizational competitive climate not only moderates the direct relationship between perceived AI usefulness and anticipated positive response but also influences the mediating role of anticipated positive response in promoting AI-related knowledge sharing behavior.

When employees perceive AI tools as useful, they tend to expect positive social feedback—such as recognition, enhanced reputation, and future rewards—which holds significant value for them. In turn, this motivates them to share AI-related knowledge. Thus, anticipated positive response serves as a crucial psychological mechanism linking perceived AI usefulness to knowledge sharing behavior.

However, this mediating mechanism depends on the organizational competitive climate. In highly competitive environments, intensified interpersonal comparison and status concerns elevate the perceived risks and personal costs of knowledge sharing, while reduced trust and reciprocity make social rewards more uncertain and less meaningful. Consequently, they perceive limited social benefit and heightened personal risk, which together weaken the motivational influence of anticipated positive feedback. Conversely, in less competitive climates characterized by trust, collaboration, and psychological safety, the perceived costs of sharing are lower and social rewards more salient. Employees are more confident that their contributions will be understood, valued, and reciprocated, thereby strengthening the mediating role of anticipated positive response in linking perceived AI usefulness to AI knowledge sharing. Accordingly, we propose the following hypothesis:

**H4.**  *Organizational competitive climate weakens the mediating effect of perceived AI usefulness through anticipated positive response in promoting AI-related knowledge sharing behavior.*

## 3. Methods

### 3.1. Participants and Procedure

The data were collected by the research team through the Credamo online survey platform, which provides access to diverse panels of employees in China. Participants were drawn from Credamo’s registered panel of working professionals, and screening criteria were applied to ensure that they were full-time employees with exposure to AI technologies in their work. The platform’s screening information indicated that all participants came from the information transmission, software and information technology services industry and the scientific research and technical services industry. These sectors represent the core domains of AI application in China, where employees are more likely to interact with AI tools in daily operations, problem-solving, and innovation processes. As such, they provide an appropriate context for examining employees’ perceptions of AI usefulness and their knowledge sharing behaviors related to AI. The sample covered a broad range of departments and job functions within these industries, providing a diverse and theoretically appropriate basis for examining the social–cognitive mechanisms underlying AI-related knowledge sharing.

Respondents received small monetary incentives provided by the platform, consistent with common practice. The use of online surveys allowed for efficiency, broad coverage, and anonymity, which helped reduce social desirability bias, although it may also have introduced self-selection bias. We implemented a three-wave survey design conducted over the course of one month, from May to June 2024, with approximately two weeks between each wave. On average, participants spent 5–7 min completing the questionnaire, indicating that the survey imposed only a minimal burden.

To ensure data quality, multiple validation and screening procedures were implemented across all three waves. Each questionnaire included at least one attention-check item (e.g., “Please select ‘Very Satisfied’ for this question”) to identify inattentive responses. Cases that failed any attention check, completed the survey in less than one-third of the median completion time, or exhibited missing or patterned responses were removed prior to analysis. These data-screening procedures helped enhance the validity and reliability of the final dataset.

**Figure 2:**
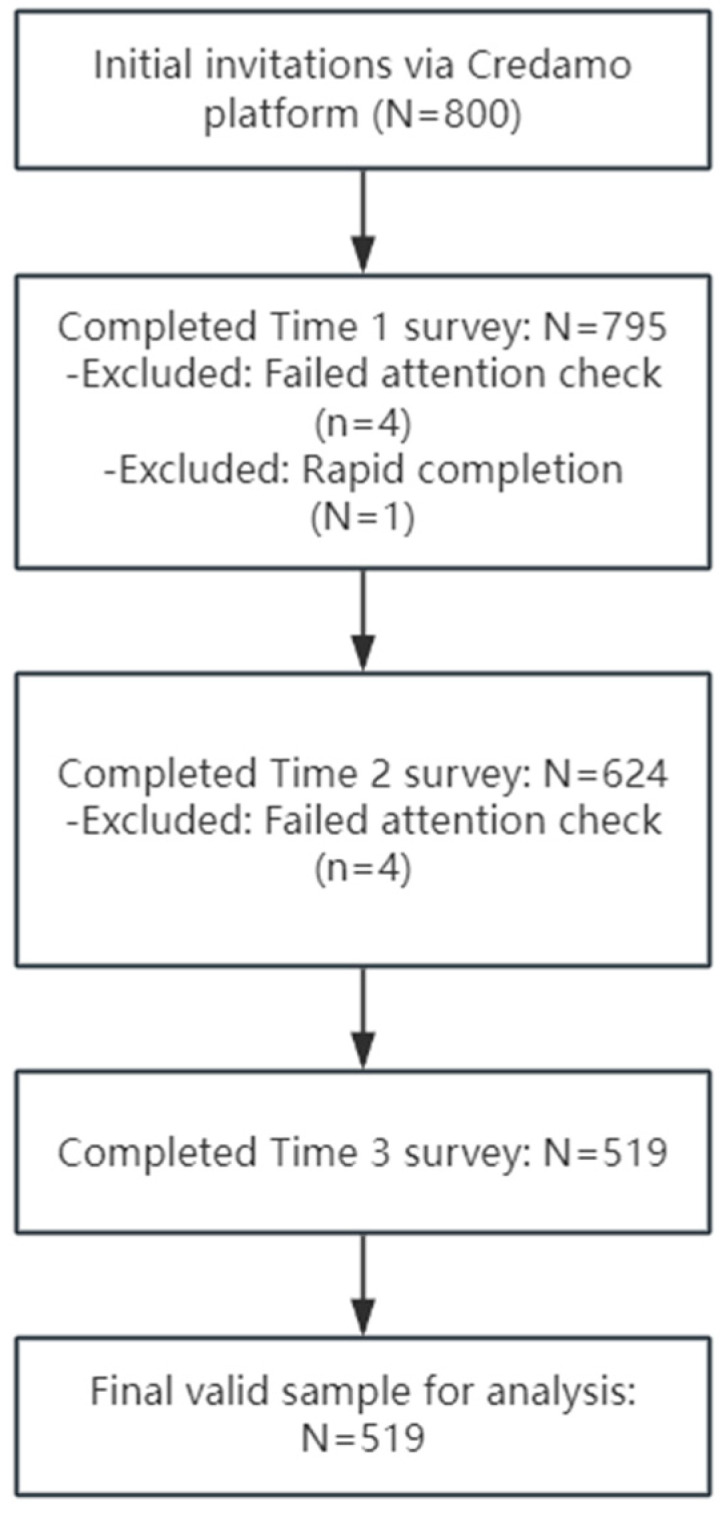
Sample Flow Diagram.

At Time 1 (T1), participants reported their perceived AI usefulness and provided demographic information. The initial sample was drawn from the Credamo platform after applying screening criteria, resulting in 795 valid questionnaires. All valid respondents were retained as a panel for subsequent follow-ups. At Time 2 (T2), the survey was sent to this panel, yielding 624 valid responses. Participants at T2 provided measures of organizational competitive climate and anticipated positive response. Following T2, the panel was further refined to include only valid respondents, who were then invited to complete the Time 3 (T3) survey. At T3, 519 valid questionnaires were collected, in which participants reported their AI-related knowledge sharing behavior. The full sample retention process across the three waves is illustrated in the Sample Flow Diagram (Figure 2).

In total, 519 employees (268 women, 51.6%) participated in all three surveys. Among them, 272 (52.4%) were under 30 years old, 490 (94.4%) were under 41, and 29 (5.6%) were between 41 and 60 years old. Before data collection, all participants were fully informed about the purpose and procedures of the study. They were explicitly told that their participation was voluntary and that they could withdraw at any time without any negative consequences. Participants were assured that all data would be collected and stored anonymously, with no personally identifiable information linked to their responses. The confidentiality of the data was strictly maintained, and responses were used solely for research purposes.

### 3.2. Measurement Adaptation and Translation

All measurement instruments in this study were adapted from well-established scales in prior research to suit the context of AI-related knowledge sharing. To ensure both linguistic and conceptual equivalence, we followed a standard translation–back translation procedure. The original English items were translated into Chinese by bilingual researchers and then independently back-translated into English by another translator. Any discrepancies were discussed and resolved through consensus to achieve semantic consistency and conceptual clarity.

Minor contextual adjustments were made to ensure relevance to AI-enabled work settings (e.g., adapting terms such as “technology” to “AI”). The preliminary Chinese version was reviewed by subject-matter experts to assess content validity and cultural appropriateness. Based on their feedback, minor revisions were made to enhance clarity and readability.

Before formal data collection, a small-scale pilot test was conducted with working professionals familiar with AI applications. Participants were asked to complete the questionnaire and provide feedback on item clarity, comprehensibility, and response difficulty. No major issues were reported. The final Chinese versions of the measures and their English sources are provided below.

### 3.3. Measures

We used 5-point Likert scales (1 = strongly disagree to 5 = strongly agree) for all key variables in this study. The original English scales were translated into Chinese using standard translation/back translation procedures (Brislin, 1986).

Perceived AI usefulness

We measured perceived AI usefulness using an adapted version of the five-item scale introduced by Choung et al. (2023). A sample item is “Using [AI virtual assistants/AI smart technologies] would enable me to accomplish tasks more quickly”. Higher scores correspond to higher levels of perceived AI usefulness. The Cronbach’s alpha coefficient for this scale was 0.80.

Anticipated positive response

We adapted the subscale of the anticipated negative and positive peer responses scale developed by Exline et al. (2004) to measure anticipated positive response in the workplace. Participants were asked, “If your colleagues knew you were using AI to complete your work, how would they feel?” They then rated their responses to a series of items on a scale that included six positive reactions. A sample item is “admiring of you”. Higher scores indicate higher levels of anticipated positive response. The Cronbach’s alpha coefficient for this scale was 0.81.

AI-related knowledge sharing behavior

We adapted a five-item scale of Connelly et al. (2012). Bavik et al. (2018) and Connelly et al. (2014) also adopted Connelly et al.’s (2012) scale to measure knowledge sharing in their studies. To fit the context of this study, the items were adapted to the AI-related domain, so that they specifically captured employees’ willingness to share knowledge concerning AI technologies. Participants were asked, “When your colleagues ask you for knowledge or information related to AI, how would you respond?” They then responded to five items used to assess the AI-related knowledge sharing behavior. Sample items include “looked into the request to make sure my answers were accurate.” Higher scores correspond to higher levels of knowledge sharing behavior. Cronbach’s alpha coefficient for this scale was 0.86.

Organizational competitive climate

We used a four-item scale, which was adapted from Fletcher et al. (2008), to assess the organizational competitive climate. A sample item is “The amount of recognition you get in this company depends on how you perform compared to others.” Higher scores indicate higher levels of organizational competitive climate. Cronbach’s alpha coefficient for this scale was 0.83.

Control variables

Because previous research has suggested that age and gender can influence knowledge sharing behavior (Lazazzara & Za, 2020; Dietz et al., 2022; Chai et al., 2011; Farooq, 2024), we controlled for age and gender statistically to reduce potential confounding effects.

### 3.4. Analytical Strategy

All data analyses were conducted using SPSS 26 and AMOS 24. First, we conducted preliminary analyses using SPSS and AMOS to examine reliability, convergent and discriminant validity, common method bias, and correlations among the variables. Then, we tested the hypotheses with SPSS PROCESS macro (version 4.1) created by Hayes (2013). Using the SPSS PROCESS macro (Model 4), we tested the direct effect of perceived AI usefulness on AI-related knowledge sharing behavior and its indirect effect through anticipated positive response, using 5000 bias-corrected bootstrap samples. After that, we further examined whether the mediation process was moderated by organizational competitive climate. The analysis of moderated mediation model was conducted using SPSS PROCESS macro (Model 7). Finally, we performed 5000-iteration bias-corrected bootstrap analysis to further examine the conditional indirect effects.

## 4. Results

We obtained valid responses from 519 participants across three survey waves. The results are presented in two stages. First, we report the preliminary analyses, including tests of reliability and validity, assessments of common method bias, and descriptive statistics. Next, we present the hypothesis testing outcomes to evaluate the proposed model and examine the robustness of the findings.

### 4.1. Preliminary Analyses

Before testing the hypotheses, we first assessed the convergent validity of the constructs. As shown in Table 1, the composite reliability (CR) values of all constructs exceeded the recommended threshold of 0.70, indicating satisfactory internal consistency. Although some average variance extracted (AVE) values were slightly below the cutoff of 0.50, their corresponding CR values were sufficiently high, suggesting that the convergent validity of the constructs remained acceptable (Fornell & Larcker, 1981).

To further examine discriminant validity, we calculated the heterotrait–monotrait (HTMT) ratios. The results in Table 2 show that all HTMT values were below the threshold of 0.85, thereby supporting adequate discriminant validity among the constructs.

In addition, a series of confirmatory factor analyses (CFAs) were conducted to compare the proposed four-factor model with several competing models (i.e., three-factor, two-factor, and single-factor models). As presented in Table 3, the four-factor model exhibited superior fit (χ^2^ = 401.775, df = 156, χ^2^/df = 2.575, TLI = 0.900, CFI = 0.918, RMSEA = 0.055, SRMR = 0.055) compared to the alternative models, confirming that the measurement model captures the distinct constructs as theorized. To further examine the robustness of the measurement structure across subgroups, a multi-group CFA was conducted by gender. The results supported configural, metric, and scalar invariance (ΔCFI ≤ 0.01, ΔRMSEA ≤ 0.015), indicating that the factor structure, loadings, and intercepts were consistent across groups (Cheung & Rensvold, 2002; Chen, 2007). These findings suggest that the measurement model operates equivalently among different respondent categories, allowing for valid comparison of structural relationships. Since age was included as a continuous control variable and its distribution was uneven across categories, additional invariance tests by age were not performed. Overall, the CFA and invariance test results confirm the adequacy, distinctiveness, and cross-group stability of the measurement model, providing a solid foundation for subsequent hypothesis testing.

Given the use of self-reported data, we also tested for common method bias (CMB) using Harman’s single-factor test on all measurement items. The test produced five factors with eigenvalues greater than 1, which reflects the number of items rather than the number of constructs. More importantly, the first unrotated factor explained only 24.5% of the total variance, well below the common 40% cutoff, suggesting that a single factor does not dominate the data. We also tested an unmeasured latent method factor model (Podsakoff et al., 2003), in which all items were loaded on both their theoretical constructs and a common latent factor. Adding this factor slightly improved the model fit (ΔCFI = 0.011, ΔTLI = 0.013, ΔRMSEA = 0.004), suggesting that the influence of common method variance was minimal. Together, these results indicate that common method bias was not a serious concern in this study.

Finally, the descriptive statistics and correlations among the study variables are reported in Table 4. Perceived AI usefulness is positively correlated with both AI-related knowledge sharing behavior (*r* = 0.41, *p* < 0.01) and anticipated positive response (*r* = 0.45, *p* < 0.01); anticipated positive response is positively correlated with AI-related knowledge sharing behavior (*r* = 0.56, *p* < 0.01).

### 4.2. Hypothesis Testing

First, using Model 4 of SPSS PROCESS macro, we tested the effect of perceived AI usefulness on AI-related knowledge sharing behavior and the mediation effect of anticipated positive response. As shown in Table 5, perceived AI usefulness is positively associated with AI-related knowledge sharing behavior (*b* = 0.50, *p* < 0.001), supporting H1. Furthermore, perceived AI usefulness is positively associated with anticipated positive response (*b* = 0.67, *p* < 0.001), and anticipated positive response is positively associated with AI-related knowledge sharing behavior (*b* = 0.38, *p* < 0.001). Then, we conducted a 5,000-iteration bias-corrected bootstrapping test, and the results showed that the indirect effect of perceived AI usefulness on AI-related knowledge sharing behavior via anticipated positive response is statistically significant (*b* = 0.25, 95% CI = [0.19, 0.33]). The mediation proportion (50.40%) was computed as the ratio of the indirect effect (0.25) to the total effect (0.50), indicating that approximately half of the total effect of perceived AI usefulness on knowledge sharing is transmitted through anticipated positive response. Therefore, H2 was supported.

**Figure 3:**
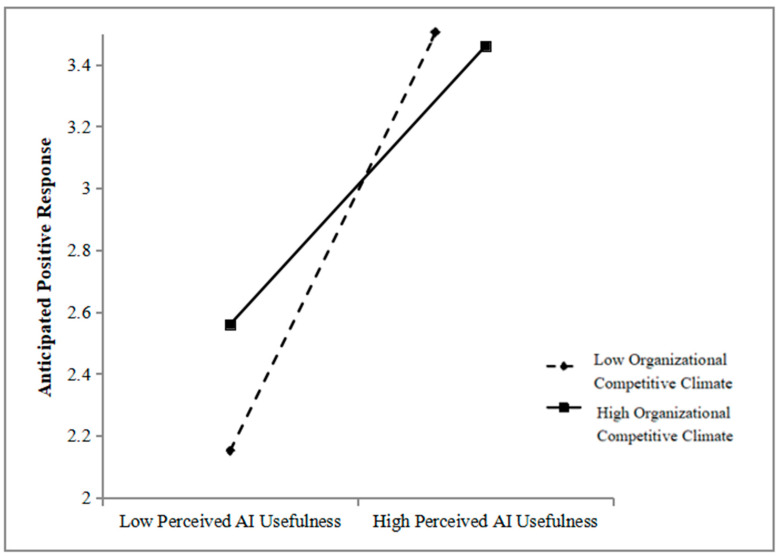
The interactive effect of perceived ai usefulness and organizational competitive climate on anticipated positive response.

Next, we included organizational competitive climate in the proposed model as the moderator. To test this moderated mediation model, we estimated the parameters using Model 7 of SPSS PROCESS macro. As Table 6 shows, the interaction term of perceived AI usefulness and organizational competitive climate was statistically significant (*b* = −0.19, *p* = 0.02), suggesting that the correlation between perceived AI usefulness and anticipated positive response was moderated by organizational competitive climate. For descriptive purposes, we plotted predicted anticipated positive response against perceived AI usefulness, separately for low and high levels of organizational competitive climate (1 SD below the mean and 1 SD above the mean, respectively), as shown in Figure 3. Simple slope tests showed that while perceived AI usefulness had a strong significant positive correlation with anticipated positive response under low organizational competitive climate (*b* = 0.82, SE = 0.09, t = 9.36, *p* < 0.001), such correlation weakened when organizational competitive climate was high (*b* = 0.47, SE = 0.11, t = 4.33, *p* < 0.001). Thus, H3 was supported.

In addition, as shown in Table 6, the control variables of gender and age had significant effects on anticipated positive response. Specifically, gender was positively associated with anticipated positive response (*b* = 0.14, *p* < 0.01), indicating that female employees tended to anticipate higher levels of positive feedback from colleagues. Age also showed a significant positive effect (*b* = 0.09, *p* < 0.05), suggesting that older employees were more likely to expect favorable social reactions. This is consistent with prior research showing that sensitivity to interpersonal relationships and social rewards varies across different demographic groups (Williams & Polman, 2015; Ng & Feldman, 2010; Kooij et al., 2011). However, neither gender nor age had a significant effect on AI-related knowledge sharing behavior.

Finally, we conducted a 5000-iteration bias-corrected bootstrap test to examine the mediation effect of anticipated positive response depending on different levels of organizational competitive climate. The results, which are shown in Table 7, indicated that the indirect effect of perceived AI usefulness on AI-related knowledge sharing behavior through anticipated positive response weakened at a high level of organizational competitive climate (*b* = 0.18, 95% CI = [0.10, 0.28]) but grew stronger at a low level of organizational competitive climate (*b* = 0.31, 95% CI = [0.22, 0.41]). The moderated mediation effect was negative and the index of moderated mediation was significant (*b* = −0.07, 95% CI = [−0.14, −0.01]). Thus, H4 was also supported.

## 5. Discussion

This study integrates the social cognitive perspective to explore how perceived AI usefulness affects AI-related knowledge sharing behavior, focusing on the mediating role of anticipated positive response and the moderating effect of organizational competitive climate. The findings indicate that perceived AI usefulness fosters knowledge sharing through its dual influence: directly by highlighting AI’s workplace relevance and indirectly by motivating employees via anticipated positive response. Additionally, the competitive climate within an organization significantly alters the strength of these relationships.

### 5.1. Theoretical Contributions

Our study enriches the research on the antecedents of AI-related knowledge sharing behavior. Prior research has identified various psychological and organizational drivers of knowledge sharing, including individual characteristics, interpersonal and team characteristics and organizational contexts (S. Wang & Noe, 2010). However, AI-driven environments have their own unique characteristics. As a rapidly evolving and transformative force, AI brings with it potential, uncertainty and complexity (Cortiñas-Lorenzo et al., 2024; Osasona et al., 2024), introducing new dynamics into knowledge sharing behaviors. By focusing on the perceived usefulness of AI technology, an extension of the TAM (Davis, 1989), our study demonstrates the significant positive effect of perceived AI usefulness on knowledge sharing behaviors. This suggests that when AI technologies are seen as genuinely helpful for enhancing work performance, they stimulate broader voluntary knowledge contributions, enriching the existing understanding of how the perceived characteristics of knowledge itself can trigger collaborative behavior.

Second, our study clarifies the unique theoretical contribution of anticipated positive response as a distinct social–cognitive mechanism within the SCT framework. Anticipated positive response captures employees’ forward-looking expectations of social recognition, appreciation, and reciprocity following knowledge sharing—expectations that differ from general intrinsic motives or material rewards. In AI-related domains, where knowledge is often both complex and controversial, employees engage in a cognitive process of weighing potential social recognition and risks that may arise from disclosure. When employees perceive AI as useful for improving their work, they are more likely to expect constructive and appreciative feedback from colleagues, which in turn enhances their motivation to share. This perspective extends prior research on general outcome expectations (e.g., Hsu et al., 2007) by specifying the social dimension of expected rewards in technology-driven settings. Moreover, our findings show that demographic characteristics subtly shape anticipated positive response: older employees and women report stronger expectations of positive feedback, suggesting that sensitivity to social rewards varies across subgroups. Within the SCT framework, these findings suggest that demographic variables influence how individuals cognitively weigh social feedback and outcome expectations, enriching the theory’s account of personal factors in behavior regulation.

Additionally, our study explores the boundary conditions of the relationship of perceived AI usefulness between knowledge sharing by introducing organizational competitive climate as a moderating factor. While earlier research has taken into account contextual elements like organizational culture and leadership (e.g., Lam et al., 2021; Abbasi et al., 2020), limited attention has been paid to the moderating impact of internal competition (J. Yu et al., 2024). The results reveal that competition functions as a social discount factor: in highly competitive climates, intensified interpersonal comparison and status anxiety elevate the perceived personal costs of knowledge sharing, while trust and reciprocity decline, making potential social rewards both less likely and less credible. Consequently, the psychological value of anticipated positive feedback is reduced, weakening its ability to motivate behavior. This insight extends SCT by clarifying how environmental cues regulate the translation of cognitive appraisals into behavior, emphasizing that social expectations are contingent on perceived relational climates.

Finally, study refines the application of social cognitive theory by identifying how cultural factors may influence the strength of its proposed mechanisms. Conducted in the Chinese organizational context, the findings suggest that collectivist cultural values—particularly the emphasis on interpersonal harmony and mianzi (Lin, 2011)—can heighten employees’ sensitivity to social evaluation, thereby strengthening the effect of anticipated positive response on knowledge-sharing behavior. This evidence indicates that the social–cognitive pathway proposed by SCT may operate differently across cultural settings, offering a more contextually grounded understanding of employee behavior in AI-integrated workplaces.

### 5.2. Methodological Contributions

In addition to its theoretical insights, this study makes several methodological contributions that strengthen the rigor and transparency of research on AI-related behavior in organizational settings.

First, by employing a three-wave time-lagged design, this study reduces potential common method bias and captures the temporal ordering among perceived AI usefulness, anticipated positive response, and knowledge sharing behavior. Although the two-week interval between survey waves cannot ensure full causal inference, it provides a reasonable temporal separation that balances internal validity with participant retention.

In addition, the study contributes to measurement transparency and validation in the AI–knowledge sharing domain. Using confirmatory factor analysis and multi-group invariance testing, we verified the discriminant validity of key constructs and reported the reliability indices in detail. This provides a clearer methodological reference for future studies developing or adapting similar constructs.

### 5.3. Practical Implications

First, our study indicates that perceived usefulness is a critical psychological trigger for voluntary knowledge exchange in digitally transforming workplaces. To foster this effect, when introducing and promoting new technologies in the workplace, organizations should focus less on top-down promotion and more on supporting employees in connecting technologies to their real work needs. This can involve offering practical, role-specific training, creating space for peer learning, and sharing grounded examples of how tools have helped colleagues solve problems or improve outcomes. Such efforts can encourage employees to see technology’s potential in their own terms, thereby fostering both the adoption and sharing of it.

Second, the mediating role of anticipated positive response suggests that organizations should focus on how employees expect the social feedback of sharing AI-related knowledge. Managers should integrate knowledge sharing into routine team interactions, such as project reviews, knowledge huddles, or learning sessions, and encourage open and friendly discussions. Leaders and supervisors should also demonstrate sharing behavior themselves, openly discussing their own practices and learnings to create psychological safety. In addition, organizations can build peer-driven recognition mechanisms, where employees naturally acknowledge those whose knowledge helped solve problems or improve workflows. Through these practices, organizations can foster a culture of openness and sharing, while encouraging the development of collaborative norms. In addition to fostering employees’ perception of AI usefulness, organizations should recognize that social and demographic diversity affects how employees respond to feedback. Designing inclusive communication and recognition systems that account for gender- and age-related differences in sensitivity to social evaluation can make knowledge-sharing initiatives more effective and equitable.

Third, our study shows that organizational competitive climate weakens the relationship between perceived AI usefulness and anticipated positive response. Specifically, this implies that an overly competitive internal environment may discourage knowledge sharing by fostering distrust and self-protection. To address this, organizations should take deliberate steps to reduce unhealthy competition and foster trust-based cultures. This may involve redesigning incentive systems and de-emphasizing individual rankings or performance metrics, shifting the focus from “winning” to “learning together.” Additionally, creating spaces for collective experimentation, such as shared AI sandboxes, cross-functional learning labs, or collaborative problem-solving sessions, can further promote a cooperative mindset. These approaches reduce the social risk of sharing and help employees feel safer contributing their knowledge in AI-driven workplaces.

Finally, as this study was conducted among employees in Chinese organizations, the findings provide context-specific insights into how cultural factors shape technology-related knowledge sharing. In collectivist workplaces, employees appear to be more responsive to peer recognition and relational feedback. For organizations operating in technology-intensive sectors, fostering open communication and mutual respect can help strengthen employees’ positive social expectations and willingness to share knowledge. Such practices not only facilitate more effective knowledge exchange but may also support the more appropriate use of emerging technologies in organizational contexts.

### 5.4. Limitations and Future Directions

Despite its valuable findings, this study has certain limitations.

First, the present study was conducted in China, a cultural context characterized by collectivist norms, strong emphasis on interpersonal harmony, and concern for mianzi (‘face’) (Lin, 2011). These cultural features plausibly increase the salience of social feedback and make anticipated positive response a particularly potent motivator for discretionary behaviors such as knowledge sharing. At the same time, this cultural specificity means the observed strength of the anticipated positive response pathway may not generalize unchanged to more individualistic contexts, where material incentives or personal achievement motives may carry relatively greater weight. Thus, we present the Chinese setting as both a theoretical asset—because it highlights how SCT’s triadic interaction operates under strong relational norms—and a boundary condition that calls for cross-cultural replication. Future studies should test the model across national and organizational cultures and include direct measures of cultural values (e.g., collectivism–individualism, face concerns) to clarify how cultural context shapes the relative importance of social versus instrumental motivators.

Second, although gender and age were statistically controlled and theoretically integrated into the framework, other potentially relevant demographic and occupational variables—such as tenure, education, or job type—were not captured due to practical constraints related to survey length and respondent burden. Future studies could employ more comprehensive datasets or multi-source designs to examine how individual and occupational characteristics influence employees’ cognitive appraisals and social expectations in technology-enabled workplaces.

Third, while the three-wave design helped mitigate common method bias and introduced temporal separation, the reliance on self-reported measures and a relatively short observation interval may constrain causal inference. This design choice reflected a balance between methodological rigor and participant retention, consistent with prior multi-wave organizational research (e.g., He et al., 2024). Nonetheless, future work could adopt longer-term longitudinal or experimental approaches to strengthen causal identification and validate the temporal dynamics proposed here.

Fourth, one limitation of our study is the convergent validity of two constructs: perceived AI usefulness and AI-related knowledge sharing behavior. The AVE values for these constructs were slightly below the conventional threshold of 0.50, although their composite reliability remained adequate. This may be due to restricted response variation for perceived AI usefulness, as most respondents rated AI usefulness consistently high, and behavioral heterogeneity for AI-related knowledge sharing behavior, since AI-related knowledge sharing involves diverse actions that do not always co-occur. We chose to retain all scale items to preserve the theoretical scope and comparability with prior research. Future studies could refine these scales, expand item sets, or apply techniques such as item parceling and multi-source data collection to further enhance convergent validity and overall measurement robustness.

Finally, as this study focused on industries with high exposure to AI technologies, the generalizability of the findings may be limited to similar technology-intensive sectors. Future research could examine organizations with varying levels of digital maturity or technological exposure to assess the robustness of the observed social–cognitive mechanisms across different industrial contexts.

### 5.5. Conclusions

By incorporating a social cognitive framework, our study shows the multifaceted ways in which perceived AI usefulness influences knowledge sharing behavior, particularly through the mediating role of anticipated positive response and the moderating effect of competitive climate. These findings enrich our understanding of the psychological and contextual factors that shape knowledge sharing behaviors in AI-enabled workplaces. They also offer actionable insights for organizations aiming to foster collaboration and innovation in the face of AI-driven transformations.

## Figures and Tables

**Table 1:** Average variance extracted and construct reliability.

	AVE	CR
Perceived AI usefulness	0.403	0.771
Anticipated positive response	0.520	0.864
AI-related knowledge sharing behavior	0.428	0.786
Organizational competitive climate	0.668	0.889

**Table 2:** Heterotrait–monotrait (HTMT) ratios of constructs.

	Perceived AI Usefulness	Anticipated Positive Response	AI-Related Knowledge Sharing Behavior	Organizational Competitive Climate
Perceived AI usefulness	—			
Anticipated positive response	0.653	—		
AI-related knowledge sharing behavior	0.668	0.738	—	
Organizational competitive climate	0.261	0.103	0.032	—

**Table 3:** Results of Confirmatory Factor Analysis.

Model	Variables	χ^2^	df	χ^2^/df	TLI	CFI	RMSEA	SRMR
Four-Factor	PAIU, APR, KSB, OCC	401.775	156	2.575	0.900	0.918	0.055	0.055
Three-Factor	PAIU, APR + KSB, OCC	502.641	159	3.161	0.863	0.885	0.065	0.060
Two-Factor	PAIU, APR + KSB + OCC	1313.252	161	8.157	0.545	0.615	0.118	0.109
One-Factor	PAIU + APR + KSB + OCC	1468.388	162	9.064	0.488	0.563	0.125	0.113

**Table 4:** Means, standard deviations, and correlations.

	M	SD	1	2	3	4	5	6
1. Gender	0.520	0.500	—					
2. Age	2.550	0.661	−0.080	—				
3. Perceived AI usefulness	4.330	0.379	−0.093 *	0.106 *	—			
4. Anticipated positive response	4.030	0.568	0.076	0.133 **	0.448 **	—		
5. AI-related knowledge sharing behavior	4.094	0.462	0.018	0.100 *	0.413 **	0.556 **	—	
6. Organizational competitive climate	3.578	0.921	−0.041	0.077	0.173 **	0.074	0.019	—

Note. N = 519; Gender coded as 0 = male, 1 = female; Age coded as 1 = below 20 years, 2 = 20–30 years, 3 = 31–40 years, 4 = 41–50 years, 5 = 51–60 years; * *p* < 0.05, ** *p* < 0.01.

**Table 5:** Results for regression analysis of mediation effect (Model 4).

	AI-Related Knowledge Sharing Behavior		Anticipated Positive Response		AI-Related Knowledge Sharing Behavior	
	M1		M2		M3	
	*b*	SE	*b*	SE	*b*	SE
Constant	1.78 ***	0.22	0.83 **	0.27	1.47 ***	0.20
Gender	0.06	0.04	0.14	0.04	0.00	0.03
Age	0.04	0.03	0.08	0.03	0.01	0.03
Perceived AI usefulness	0.50 ***	0.05	0.67 ***	0.06	0.25 ***	0.05
Anticipated positive response					0.38 ***	0.03
R^2^	0.18 ***		0.22 ***		0.34 ***	
ΔF	36.98 ***		49.52 ***		67.00 ***	

Note: N = 519. ** *p* < 0.01, *** *p* < 0.001.

**Table 6:** Results for regression analysis of moderated mediation effect (Model 7).

	Anticipated Positive Response			AI-Related Knowledge Sharing Behavior		
	*b*	SE	95% CI	*b*	SE	95% CI
Constant	3.75 ***	0.09	[3.57, 3.95]	2.55 ***	0.14	[2.27, 2.83]
Gender	0.14 **	0.04	[0.05, 0.23]	0.00	0.34	[−0.06, 0.07]
Age	0.09 *	0.03	[0.02, 0.15]	0.01	0.03	[−0.04, 0.06]
Perceived AI usefulness	0.65 ***	0.06	[0.53, 0.75]	0.25 ***	0.05	[0.15, 0.35]
Organizational competitive climate	0.01	0.03	[−0.04, 0.06]			
Perceived AI usefulness × Organizational competitive climate	−0.19 *	0.08	[−0.36, −0.03]			
Anticipated positive response				0.38 ***		
R^2^	0.23 ***			0.34 ***		
F	31.00 ***			67.00 ***		

Note: N = 519. * *p* < 0.05, ** *p* < 0.01, *** *p* < 0.001.

**Table 7:** Bootstrapping results for conditional indirect effect (Model 7).

Conditional Indirect Effect at High and Low Levels of Organizational Competitive Climate	Effect	SE	95% CI
−1 SD (−0.92) organizational competitive climate	0.31	0.05	[0.22, 0.41]
Mean (0) organizational competitive climate	0.24	0.04	[0.18, 0.32]
+1 SD (0.92) organizational competitive climate	0.18	0.05	[0.10, 0.28]
Index of Moderated Mediation	−0.07	0.03	[−0.14, −0.01]

Note: N = 519; bootstrap size = 5000.

## Data Availability

The data supporting the findings of this study are available from the corresponding author upon reasonable request.
